# Spectrofluorometric quantitative analysis of aripiprazole based on quenching of natural derived carbon quantum dots in spiked human plasma

**DOI:** 10.1038/s41598-023-47392-2

**Published:** 2023-11-29

**Authors:** Saleh I. Alaqel, Arwa S. Alqahtani, Adnan Alharbi, Yusuf S. Althobaiti, Ahmed K. Bamaga, Majed A. Algarni, Ahmed A. Almrasy, Atiah H. Almalki

**Affiliations:** 1 Department of Pharmaceutical Chemistry, Faculty of Pharmacy, Northern Border University, Rafha, 91911 Saudi Arabia; 2 Department of Chemistry, College of Science, Imam Mohammad Ibn Saud Islamic University (IMSIU), P.O. Box 90950, Riyadh, 11623 Saudi Arabia; 3 Clinical Pharmacy Department, College of Pharmacy, Umm Al-Qura University, Makkah, Saudi Arabia; 4 Addiction and Neuroscience Research Unit, Health Science Campus, Taif University, P.O. Box 11099, Taif, 21944 Saudi Arabia; 5 Department of Pharmacology and Toxicology, Taif University, P.O. Box 11099, Taif, 21944 Saudi Arabia; 6 Neurology Division, Pediatric Department, Faculty of Medicine, King Abdulaziz University Hospital, King Abdulaziz University, Jeddah, Saudi Arabia; 7 Department of Clinical Pharmacy, College of Pharmacy, Taif University, P.O. Box 11099, Taif, 21944 Saudi Arabia; 8 Pharmaceutical Analytical Chemistry Department, Faculty of Pharmacy, Al-Azhar University, 11751 Nasr City, Cairo, Egypt; 9 Department of Pharmaceutical Chemistry, College of Pharmacy, Taif University, P.O. Box 11099, Taif, 21944 Saudi Arabia

**Keywords:** Chemistry, Materials science

## Abstract

Autism spectrum disorder is a significant concern worldwide, particularly in Middle Eastern countries. Aripiprazole, a psychiatric medicine that works as a partial agonist at D_2_ receptors, is often used for autism-related behavior issues in children. Monitoring the therapy of aripiprazole could enhance the safety and effectiveness of treatment for autistic individuals. The purpose of this study was to develop a highly sensitive and environmentally friendly method for analysis of aripiprazole in plasma matrix. To achieve this, water-soluble N-carbon quantum dots were produced from a natural green precursor, guava fruit, and used in fluorescence quenching spectroscopy to determine the presence of aripiprazole. The synthesized dots were analyzed and characterized using transmission electron microscopy and Fourier transform infrared spectroscopy, and they showed a strong fluorescence emission peak at 475 nm. The proposed method was validated according to ICH M10 guidelines and was shown to be highly sensitive, allowing for nanoscale determination of aripiprazole in plasma matrix. Additionally, the method was compared to a previously reported spectrophotometric method, and it was found to be more sensitive and consistent with the principles of green analytical chemistry.

## Introduction

Autism, also known as autism spectrum disorders (ASD), refers to a group of neurological and developmental conditions^1–5^. The prevalence of autism has increased globally, particularly in the Arab region, due to greater social awareness campaigns, reduced cultural stigma, and the availability of expert medical professionals^6–12^. Although there are no specific medications that target the core symptoms of autism, atypical antipsychotics such as aripiprazole and risperidone can be used to treat associated symptoms like irritability^13^.

**Figure 1:**
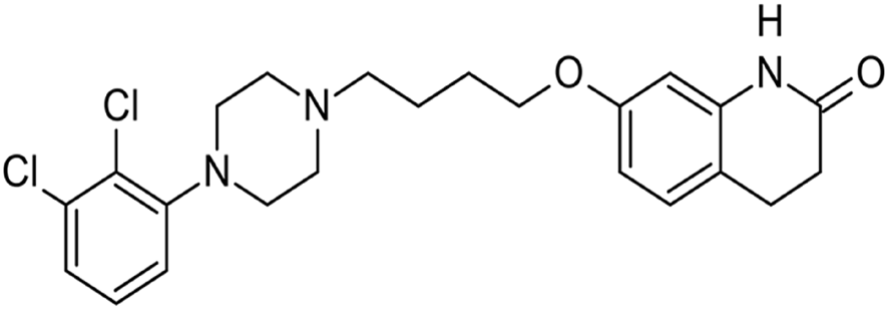
Structural formula of ARP.

Aripiprazole, depicted in Fig. 1, is a psychotropic medication that acts as a partial agonist at D_2_ receptors. It is prescribed for the treatment of schizophrenia, manic depression, Tourette syndrome, and irritability linked with autism in children and adolescents^14^. However, its usage in this population has been linked with notable adverse effects, including sedation, weight gain, extrapyramidal effects, hyperprolactinemia, and metabolic irregularities^15^. Therefore, it is crucial to monitor patients clinically to assess the occurrence of adverse effects that could compromise the benefit-risk ratio^16^.

Several techniques have been reported for detecting ARP, such as separation, spectroscopic, and electrochemical methods. Separation techniques include HPLC, LC-MS/MS, capillary electrophoresis, and HPTLC approaches, while the electrochemical techniques involve adsorptive stripping and linear scan voltammetry^17–34^. These techniques are commonly employed to analyze drugs in various matrices and achieve ultra-sensitive detection, but they come with certain drawbacks such as lengthy analysis time, the need for sample preparation, high cost, and limited availability in small laboratories. Additionally, they are not in line with the principles of green analytical chemistry, as they consume high levels of energy, generate more waste, and require the use of hazardous chemicals. Various direct UV spectrophotometric measurements and mathematical UV spectrophotometric manipulation methods have been reported to determine ARP^35–39^. In addition, several indirect spectrophotometric approaches based on chemical derivatization processes have been reported^40–46^. Although simple and cost-effective, these methods lack the ability to detect ARP in biological samples and involve the use of toxic chemicals that are not environmentally friendly. Another technique, which utilizes an indirect spectrofluorimetric approach, has been documented in literature. This technique involves the reaction of ARP with o-phthalaldehyde in the presence of 6-mercaptoethanol in a borate buffer of pH 9.0 or pH 10^47^. However, the sensitivity of this method is inadequate for detecting the drug in biological samples.

The basic objective of this research is to establish an analytical method for measuring ARP in plasma samples that is accurate, simple, and environmentally friendly, with an exceptionally sensitive detection limit.

Recently, carbon quantum dots (CQDs) have gained significant attention as fluorescent probes for detecting drugs in various sample matrices^48–56^. In comparison to those obtained from chemical precursors, naturally derived CQDs, particularly those derived from herbal medicine, offer several benefits. These benefits include biological compatibility, broad availability, environmental friendliness, and a high content of affordable and renewable raw materials including C, H, N, and O atoms, such as polysaccharides, proteins, nucleic acids, and phospholipids.

In this work, a novel water-soluble N-CQDs was synthesized from guava fruit as a natural fluorescent probe. The synthesized probe was applied for quantifying ARP in spiked human plasma based on the quenching characters of ARP. The method was highly sensitive with a lower limit of detection (LLOQ) of 4 ng/mL. The given study differs from prior publications in that it provides a simple and eco-friendly procedure with a very low detection limit capable of monitoring ARP in plasma without the need of costly separation procedures, and toxic chemicals. The fabricated method could be significantly applied in therapeutic monitoring of ARP in the autistic patients to assess the occurrence of adverse effects that could compromise the benefit-risk ratio. The sustainability of the applied method was assessed using the analytical eco-scale and analytical greenness metric (AGREE) and the results confirmed the sustainability of the method.

## Experimental

### Materials and chemicals

Pure ARP powder was provided by Al Andalous for the pharmaceutical industry, Egypt. Human plasma samples from the National Egyptian Blood Bank were utilized for the analysis. The chemicals utilized in the analysis were of analytical grade and high purity, sourced from Egypt's El Nasr Company. The water employed in the procedure was recently double distilled. The guava fruits utilized in the study were procured from a local Egyptian market. Buffer solutions with varying pH levels were created in accordance with the guidelines set out in the US Pharmacopoeia.

### Instrumentation

Fluorescence measurements were performed using a Jasco FP-6200 Spectrofluorometer (Japan) equipped with 150-Watt Xenon lamp. Slit widths for both monochromators were set at 10 nm. A 1 cm quartz cell was used. UV- Visible measurements were performed using a Shimadzu UV–Visible 1800 Spectrophotometer (Japan), equipped with 10 mm matched quartz cells. Images of transmission electron microscopy (TEM) were acquired using a JEOL JEM -M2100 transmission electron microscope set at 200 kV (USA). Fourier transform infrared (FTIR) spectrum was conducted on a PerkinElmer FTIR spectrophotometer (USA).

### Standard solutions

To prepare a standard stock solution of ARP, 10 mg of drug powder was transferred into a 100-mL volumetric flask containing 20 mL of ethanol and 40 mL of water. The mixture was shaken until the powder dissolved and then the flask was filled with water to the mark. This resulted in a stock solution with a concentration of 100 μg/mL. From this stock solution, a working solution with a concentration of 1 μg/mL was made by diluting it further with water.

### Synthesis of N-CQDs

The guava fruit was sliced into pieces and the pulp and seeds were removed before it was carbonized in an electric oven at a temperature of 250 °C for 50 min for a duration of 50 min. Once cooled, the carbonized guava product was ground into a fine powder. A 200-mg equivalent of the powder was then mixed in 60 mL of freshly double-distilled water and boiled for 10 min. After boiling, the mixture was centrifuged for 20 min and the resulting yellow solution was filtered into a 100-mL volumetric flask. The flask was then filled to the desired volume with double-distilled water.

### Method development and validation

The calibration standards for ARP were created by adding specific amounts of the working solution (ranging from 40 to 1600 ng) into centrifuge tubes containing 1 mL of human plasma and 2 mL of acetonitrile. The tubes were vortexed for 1 min before being centrifuged at 5000 rpm for 20 min. The generated supernatants were then evaporated to dryness in a rotary evaporator and the residues were dissolved in ethanol and transferred to 10-mL volumetric flasks. To each flask, 0.5 mL of N-CQDs solution (having 1000 µg N-CQDs) and 1 mL of borate buffer solution (pH 8) were added, mixed well, and then the flasks were adjusted to the desired volume with water. During the calibration curve development, the lower limit of quantification (LLOQ) was set at 4 ng/mL and the upper limit of quantification (ULOQ) was set at 160 ng/mL. A blank sample was also made under the same conditions. The fluorescence intensities of the calibration samples (F) and the blank sample (F0) were measured at 475 nm after excitation at 380 nm, and the calibration curve was constructed by plotting (F0/F) against the drug concentrations in ng/mL.

For validation studies, quality control (QC) samples were prepared at four different levels: LLOQ, lower quantifiable concentration (LQC), middle quantifiable concentration (MQC), and high quantifiable concentration (HQC). The QC samples were prepared at concentrations of 4 ng/mL for LLOQ, 10 ng/mL for LQC, 70 ng/mL for MQC, and 130 ng/mL for HQC. A set of five replicates of QC samples were analyzed on the same day for intra-day accuracy and precision, and on three different days for inter-day accuracy and precision. The matrix effect was evaluated efficiently by analyzing three replicates of LQC and HQC samples. The CS and QC samples were created by adding ARP aliquots to pooled plasma from different sources.

## Results and discussion

### Characterization of N-CQDs

**Figure 2:**
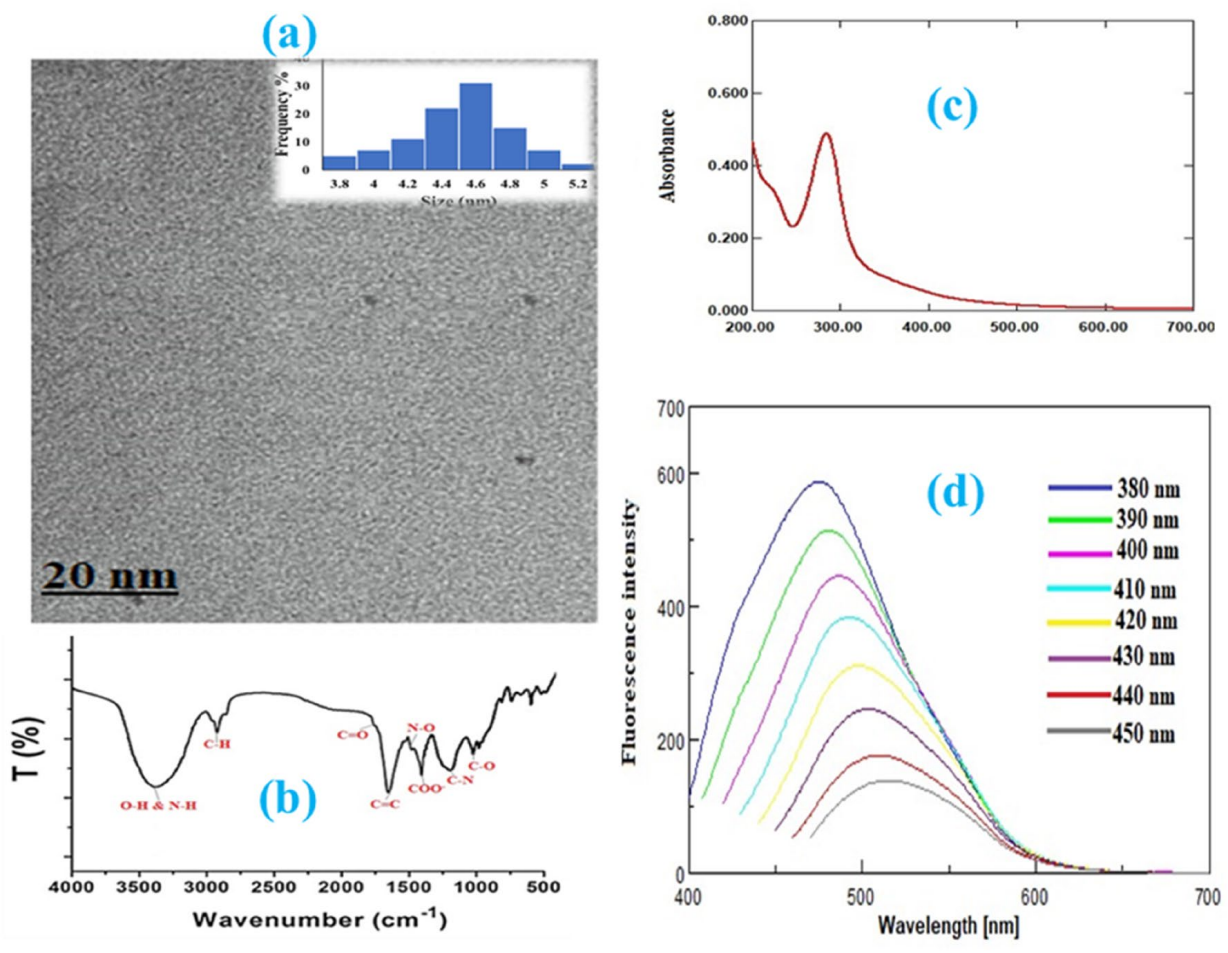
(**a**) TEM image and diameter distribution of N-CQDs, (**b**) FTIR spectra of N-CQDs, (**c**) UV–vis absorption spectra of N-CQDs, and (**d**) excitation dependent emission spectra of N-CQDs.

The transmission electron microscope (TEM) was used to examine the morphology and size of N-CQDs. The synthesized N-CQDs was observed to have a uniform spherical shape, with a diameter distribution ranging from 3.8 to 5.2 nm with an average diameter of 4.5^54,55^. Fourier transformed infrared (FT-IR) spectroscopy was used to study the functional groups on N-CQDs. The peaks observed at different wave numbers were attributed to various groups such as C–H, C=O, C=C, N–O, COO–, C–N, C–O, O–H, and N–H. Specifically, a broad peak at 3376 cm^-1^ was related to O–H and N–H groups (see Fig. 2b)^55,57^.

### Spectral properties of N-CQDs

UV–vis and fluorescence spectroscopy were employed to study the spectral properties of N-CQDs. The UV–vis absorption spectra of N-CQDs showed a primary peak at 283 nm with an extension into the visible range, indicating the electronic transition of the C=C and C=O bands (see Fig. 2c)^54^. Additionally, N-CQDs emitted a powerful fluorescence at 475 nm upon excitation at 380 nm. The excitation-dependent property of N-CQDs was examined by altering the excitation wavelength from 380 to 450 nm. The emission fluorescence peak shifted to 515 nm and had a significant reduction in intensity when the excitation wavelength was increased, which confirmed the excitation-dependent emission property of N-CQDs, a defining feature of CQDs (see Fig. 2d)^54,57^.

The quantum yield of N-CQDs was calculated using a comparative single-point method with quinine sulfate as a fluorescence reference standard in 0.01 M H_2_SO_4_ at an excitation wavelength of 380 nm according to the following equation^58,59^:

1 $${\varphi }_{s =} {\varphi }_{r }\left(\frac{{A}_{r}}{{A}_{s}}\right) \left(\frac{{E}_{s}}{{E}_{r}}\right) \left(\frac{{\eta }_{s}^{2}}{{\eta }_{r}^{2}}\right)$$

Where *Φ* is fluorescence quantum yield, *η* is a refractive index of the solvent, *A* is absorbance of the solution, *E* is integrated fluorescence intensity (area) of the emitted light, and subscript “*r”* and “*s”* refer to the reference and sample, respectively. The quantum yield of guava fruit N-CQDs was calculated to be 26.12%.

### Mechanism of quenching

**Figure 3:**
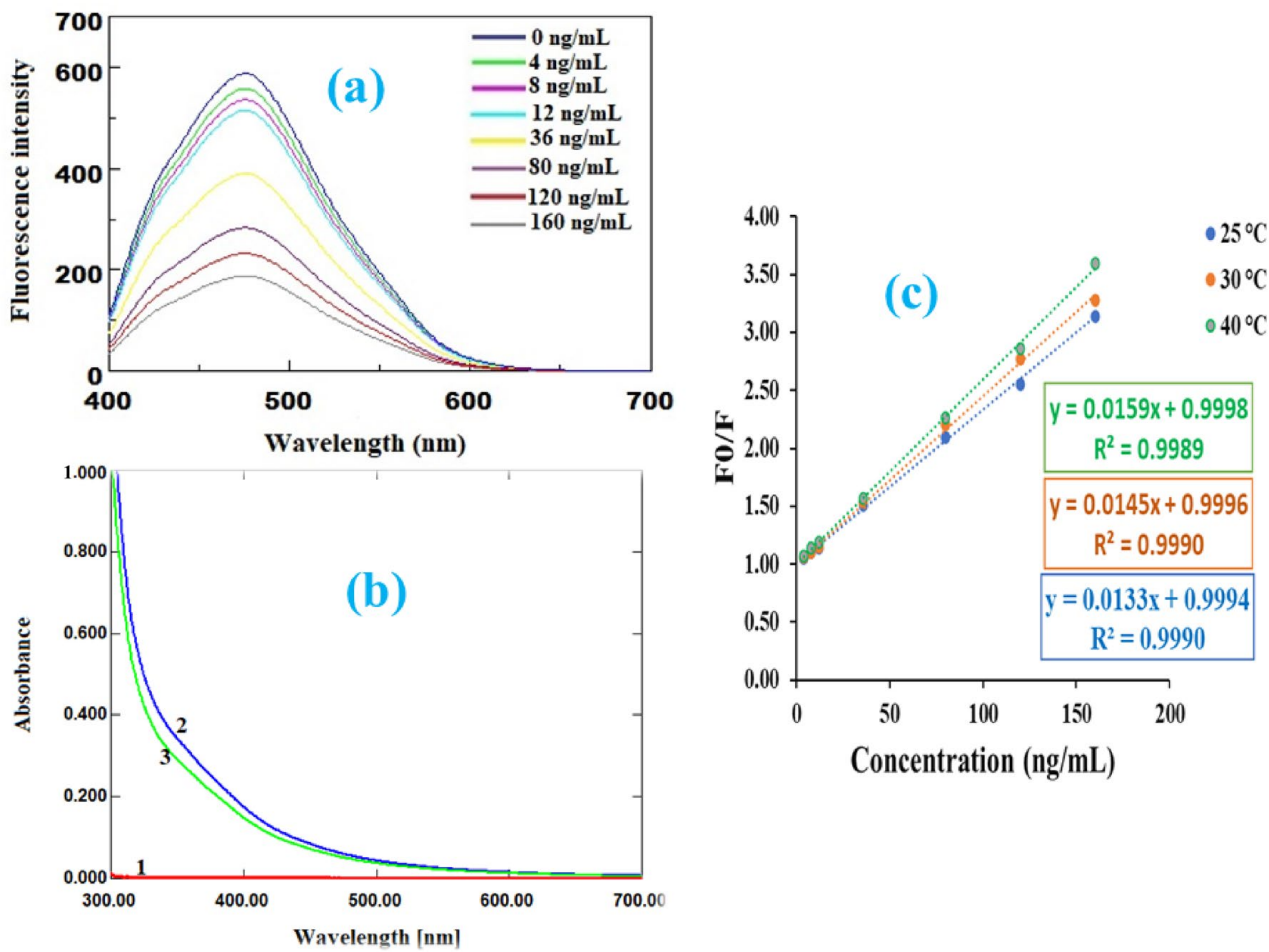
(**a**) Quenching reaction of N-CQDs with different concentrations of ARP; (**b**) Absorption spectra of ARP (1), and N-CQDs before (2) and after (3) the addition of ARP; (**c**) Stern–Volmer plot at different temperature.

The fluorescence intensity of N-CQDs gradually decreases as the concentration of ARP increases, as shown in Fig. 3a, demonstrating that fluorescence quenching is concentration-dependent.

After comparing the absorption spectra of ARP, N-CQDs before addition ARP, and N-CQDs after addition ARP (Fig. 3b), it is clear that ARP has no absorption responses in the 300–600 nm wavelength region. As a result, the quenching impact of ARP was not caused by the inner filter effect of ARP absorption, but rather by the interaction between ARP and the functional groups on the N-CQDs surface. If the quencher forms a non-fluorescent complex with N-CQDs in the ground state, the quenching is static, whereas dynamic quenching occurs when the quencher collides with N-CQDs in the excited state^60,61^. To determine whether the quenching mechanism of ARP-NCQDs interaction is dynamic or static, the calibration curve was plotted (between *F*_*0*_*/F* and *Q*) at different temperatures (25°°C, 30 °C, and 40 °C) using the Stern–Volmer equation^62,63^:

2 $$\frac{{F_{0} }}{F} = 1 + K_{SV} \left\lceil Q \right\rceil$$

where *F*_*0*_ and *F* are the fluorescence intensities of CQDs in the absence and presence of ARP, *K*_*SV*_ is the Stern–Volmer quenching constant (slope), and *Q* is the ARP concentration.

In dynamic quenching, the slope (Stern–Volmer quenching constant) increases with increasing temperature, whereas in static quenching, the slope decreases with increasing temperature. As shown in Fig. 3c, the slope value increased as temperature increased, implying that the quenching mechanism of ARP-NCQDs interaction is dynamic.

### Method optimization

**Figure 4:**
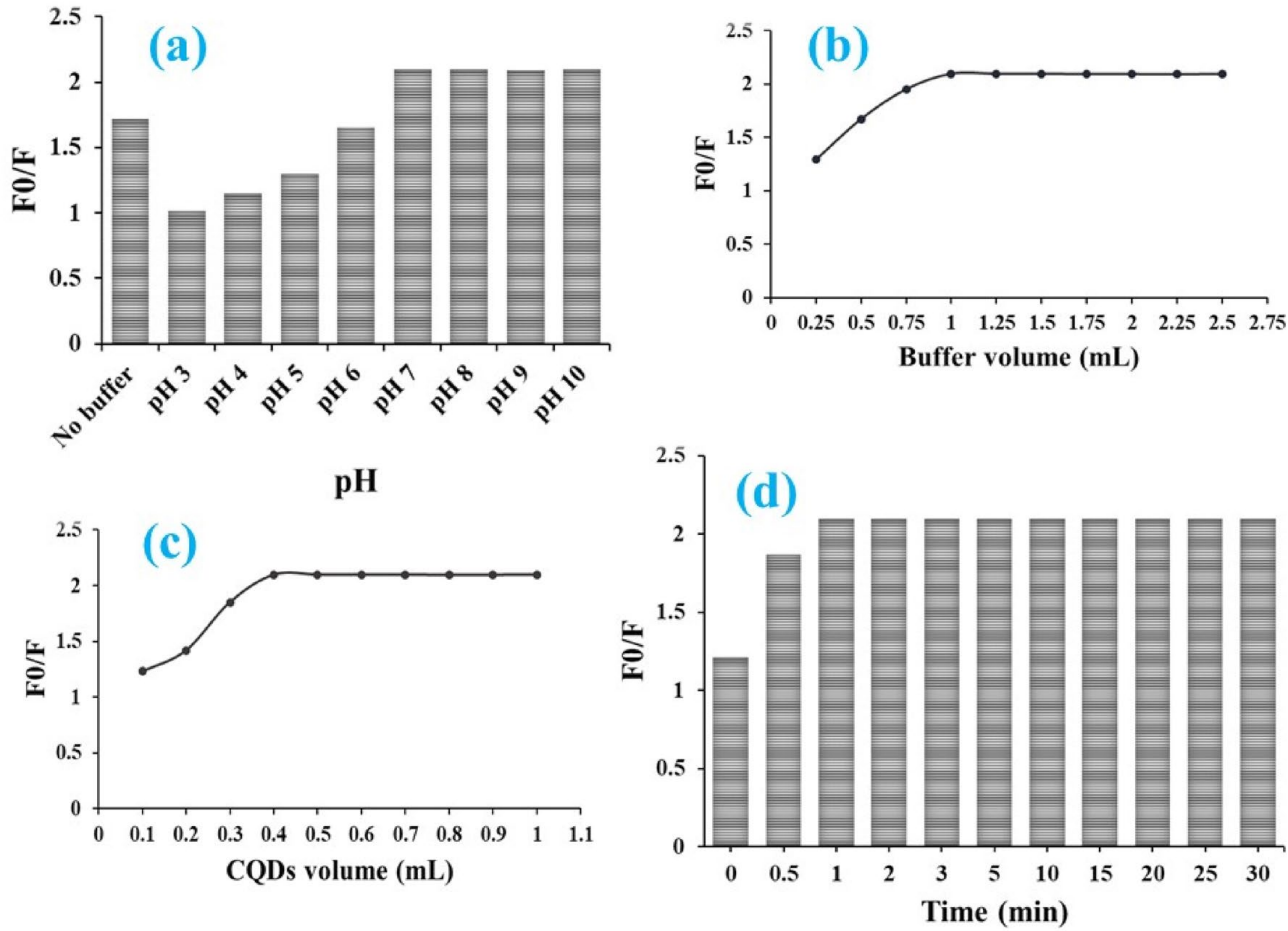
Optimization of experimental parameters influencing the stability of fluorescence quenching of N-CQDs by ARP including (**a**) buffer pH, (**b**) buffer volume, (**c**) N-CQDs volume, and (**d**) incubation time.

The stability of fluorescence quenching of N-CQDs by ARP was optimized by investigating various factors. These factors included the pH and volume of the buffer, the volume of N-CQDs and the incubation time. The fluorescence quenching was assessed with and without the addition of buffer solutions with pH values ranging from 3 to 10. As indicated in Fig. 4a, steady quenching was obtained using a pH 8 borate buffer solution. The quenching reaction substantially decreases as the solution's acidity increases. Different volumes of buffer solution were examined, and it was found that 1 mL is optimal (Fig. 4b). Furthermore, fluorescence quenching was studied using various volumes of N-CQDs solution. It was discovered that 0.5 mL of N-CQDs solution containing 100 µg/mL N-CQDs resulted in the best fluorescence quenching with the drug (Fig. 4c). The efficiency of fluorescence quenching in the presence of ARP was investigated at varying time intervals ranging from 0 to 30 min. The maximum fluorescence quenching of N-CQDs was obtained within 2 min, and no further quenching was observed as the reaction time increased (Fig. 4d).

### Method validation

The ICH M10 guidelines were used to validate the method, which included assessing its linearity, selectivity, accuracy, precision, and matrix effect. The method demonstrated excellent linearity over seven calibration standard levels in the concentration range of 4–160 ng/mL, with a coefficient of determination of 0.9990 (Table 1). The method was highly sensitive, with an LLOQ of 4 ng/mL and a ULOQ of 160 ng/mL. The selectivity of the method was confirmed by analyzing a blank sample spiked by pooled plasma sample from different sources, which did not interfere with the N-CQDs emission. The accuracy and precision of the method were evaluated by analyzing QC samples on the same day and on three different days and found to be accurate and precise with %CV ranging from 0.65 to 5.01% and from 1.79 to 4.63% for intra-day and inter-day, respectively, and with accuracy ranging from 95.19 to 102.48% and from 96.49 to 99.71% for intra-day and inter-day, respectively (Table 2). The matrix effect was also tested by analyzing three replicates of LQC and HQC samples spiked with plasma from different sources, and the method was found to be unaffected by endogenous components of plasma matrix for the drug at LQC and HQC concentrations. The precision and accuracy for ARP at LQC concentration were determined to be 2.72% and 96.58%, respectively, while at HQC concentration, they were 2.09% and 98.75%.

**Table 1:** Regression and validation data for the determination of ARP using N-CQDs.

Parameters	ARP
Excitation wavelength (nm)	380
Emission wavelength (nm)	475
Linearity range (ng/mL)	4 − 160
LLOQ (ng/mL)	4
ULOQ (ng/mL)	160
Slope	0.0133
Intercept	0.9994
Coefficient of determination (r^2^)	0.9990

**Table 2:** Accuracy and precision of the assay for ARP in plasma (n = 5).

Level	Added concentration (ng/mL)	Analysis time	Concentration found (mean ± SD, ng/mL)	Accuracy (mean%)		Precision (%CV)	
				Intra-day	Inter-day	Intra-day	Inter-day
LLOQ	4	Day 1	4.10 ± 0.19	102.48	99.71	4.70	4.63
		Day 2	3.91 ± 0.20	97.74		5.01	
		Day 3	3.96 ± 0.14	98.91		3.53	
LQC	10	Day 1	9.77 ± 0.34	97.74	96.49	3.51	2.79
		Day 2	9.65 ± 0.25	96.53		2.64	
		Day 3	9.52 ± 0.18	95.19		1.85	
MQC	70	Day 1	67.41 ± 1.56	96.31	97.23	2.31	2.44
		Day 2	69.22 ± 1.68	98.88		2.43	
		Day 3	67.55 ± 1.36	96.50		2.02	
HQC	130	Day 1	127.43 ± 0.83	98.02	99.47	0.65	1.79
		Day 2	131.56 ± 1.43	101.20		1.09	
		Day 3	128.93 ± 2.25	99.18		1.75	

### Selectivity experiment

Selectivity of the fabricated method for ARP was examined in the presence of various interfering species. Metal ions and biomolecules that are increased in autism are among these interfering species. An illustration of the interfering species, its concentration, and the percentage change in quenching is presented in Table 3. The results in Table 3 showed that the fluctuation in quenching% for all interfering species was less than 5%, although the interfering species concentrations used were much higher than the greatest level reported in blood. The findings demonstrated that the technique was selective for ARP.

**Table 3:** Selectivity studies in the presence of various interfering species.

Interfering species	Conc. of interfering species (µg/mL)	Quenching %
Hg^2+^	0.011	1.86
Al^3+^	0.045	2.40
Cu^2+^	8.145	0.73
Glutamate	6.787	3.81
Glycine	41.628	2.74
Glutamine	82.578	4.05
Urea	3339.6	0.58
Albumin	791.84	2.39

### Stability experiment

**Figure 5:**
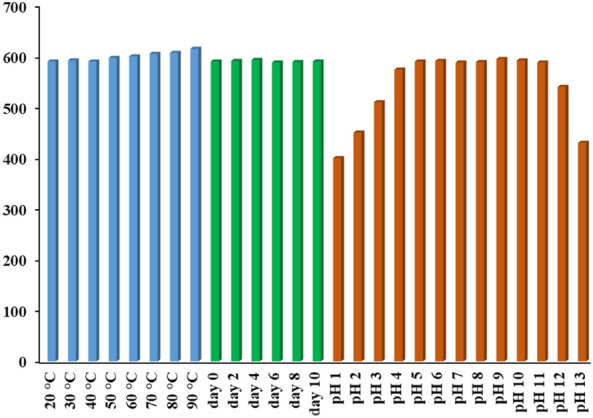
Stability studies of N-CQDs under different thermal, storage duration, and pH conditions.

The thermal stability, storage duration stability, and pH stability of the synthesized N-CQDs were evaluated. Thermal stability was investigated across a wide range of heating temperatures ranging from 20 to 90 °C. The fluorescence intensity of the N-CQDs remained steady at all temperatures, as shown in Fig. 5, with negligible increase at high temperatures. The storage duration stability of N-CQDs, on the other hand, was studied over a 10-day period at varying day intervals. The fluorescence intensity of the N-CQDs remained steady over this period, as illustrated in Fig. 5. The stability of the N-CQDs was also investigated throughout a pH range of 1–13. As demonstrated in Fig. 5, the fluorescence intensity of N-CQDs remained nearly steady throughout a wide pH range of 5–11, showing the N-CQD's potential in a variety of fields^64^.

### Comparison with the previous published work

The proposed method was compared to a previously reported spectrophotometric method^38^. The suggested method was found to be more sensitive and allowed for Nano detection of the drug in different matrix samples with detection limit and linearity ranges of 4 ng/mL and 4–160 ng/mL, respectively, compared to 0.26 µg/mL and 5–30 µg/mL for the reported method as shown in Table 4. The suggested method was also evaluated based on its adherence to green analytical chemistry principles using two metrics, namely, the analytical eco-scale and analytical greenness metric (AGREE)^65–68^. The results showed that the suggested method was greener than the reported method in terms of both metrics, with a total score of 95 for the analytical eco-scale compared to 87 for the reported method and a higher AGREE score of 0.78 (green) compared to 0.67 (yellowish green) for the reported method as shown in Table 4.

**Table 4:** Comparison between the proposed and the reported methods.

Parameters	The proposed method		The reported method^38^	
Sensitivity	4 ng/mL		0.26 µg/mL	
Linearity range	4–160 ng/mL		5–30 µg/mL	
Matrix	Plasma		Pharmaceutical	
Greenness assessment	Analytical eco-scale			
	Parameters	Penalty points	Parameters	Penalty points
	Reagents		Reagents	
	Water	0	Methanol	6
	Borate buffer pH 8	2	HCl	4
	Instrument spectrofluorometer		Instrument spectrophotometer	
	Energy: < 0.1kWh per sample	0	Energy: < 0.1kWh per sample	0
	Occupational hazards	0	Occupational hazards	0
	Waste (1–10 mL)	3	Waste (1–10 mL)	3
	**∑** Penalty points	**5**	∑ Penalty points	**13**
	Total scores	**100–5 = 95**	Total scores	**100–13 = 87**
	AGREE tool			
	

Significance values are in bold.

## Conclusion

In this study, N-carbon quantum dots were synthesized from guava fruit and utilized for analysis of aripiprazole through the phenomenon of fluorescence quenching spectroscopy. The synthesized quantum dots exhibited a strong fluorescence peak at 475 nm when stimulated at 380 nm, and their fluorescence intensity was inversely proportional to the concentration of aripiprazole. This method was successfully employed to determine aripiprazole content in spiked human plasma. The proposed technique demonstrated higher sensitivity for nanoscale detection and adhered better to the principles of green analytical chemistry compared to a previously reported spectrophotometric method.

### Ethics approval and consent to participate

This work was approved by the Committee of Research Ethics in the Faculty of Pharmacy, Al-Azhar University, Cairo, Egypt. All participants signed an informed consent statement before participation in the study. All described procedures were performed in accordance with relevant guidelines and regulations**.**

## Data Availability

The datasets used and/or analyzed during the current study are available from the corresponding author on reasonable request.
